# Looking Forward More Truly

**Published:** 1890-02

**Authors:** E. D. Babbitt

**Affiliations:** 50 Union Square, N. Y.


### LOOKING FORWARD MORE TRULY.

BY E. D. BABBITT, M. D.

So completely has Mr. Preston befogged the subject of co-operation and colonization that I must necessarily say something further on the subject. In fact, he becomes doubly animated in condemning methods of human upbuilding, and sneers at those persons who are making efforts to place society on a nobler basis. I believe that he has not made one statement in this last article but what is misleading.

In the first place he goes on to bring up the case of the Botany Bay Colony of villians and criminals who were banished from England and sent to Australia as a punishment for their crimes. He says that "Botany Bay has generally been regarded as a good place to keep away from," hence by inference he would signify that all colonization is bad. Bright reasoning that.

He then refers to the Credit Foncier Company which is colonizing Topolobampo Bay on the Gulf of California, in Mexico. He speaks of "a few half-famished folks rusticating in ragged tents;" "houseless hungry tent dwellers," and makes his insinuations about "gathering up the destitute, depraved and distressed and making them useful members of co-operative communities," and of putting the "Bowery bum" down at Pacific City, etc.

Now what is the truth of the matter? Some of the noblest and purest souls that walk this earth are members of that colony. Mr. A.

K. Owen, the founder, has sifted the condition of the nations of the world, has written very able treatises on co-operation which different colonies are patterning after, and has worked some fourteen years without salary to perfect and advance the colony. The sneer that he remains here is in part correct, as he has to arrange for capital to complete his purposes in Sinaloa. One other director, John W. Lovell, the eminent publisher and one of the truest souled men in New York city, also remains here, and should remain here, at least for the present, as he can do more good by carrying on his great business. This Mr. Lovell is the treasurer of the colony. Another leading member is Mrs. Marie Howland, the editor of the colony paper and the author of the work that should make her name immortal, namely, "Papa's Own Girl," one of the most ennobling works ever written. Jesse Grant owns stock in this company and so does Ex-Governor Rice, and a number of the people already on the ground, nearly 200 in number, at Topolobampo and La Logia, are among the choice people of the earth. They are raising most of their own produce, live on a soil of wonderful fertility and in a climate of remarkable beauty and healthfulness. They have their schools, their Lyceum, their happy social gatherings; have a number of houses already completed, and although they have deprivations are contented and harmonious. Topolobampo Bay is one of the finest harbors in the whole world and must become the center of a great shipping trade. The railroad from Galveston to Topolobampo was projected by General Grant and his friends, and the Mexican government has made great concessions in its favor. When Mr. Preston calls the Credit Foncier Co., "a community in Mexico incorporated under the statutes of one of our States," he misrepresents. It is an American colony, doing an extensive business in the United States, organized in the United States and capitalized here. Its connection with Mexican interests has been arranged by eminent lawyers, and I think they know what they are about as well as Mr. Preston, who has only skimmed the surface of things. If they fail to carry out their grand purpose by not being able to complete the railroad which they have begun, or finish paying for Topolobampo and vicinity, it will certainly be due to a great extent, to the opposition of such writers as Mr. Preston, and to those newspapers which have constantly falsified the whole movement. When the *Sun* gave a friendly account of the colony, a newspaper here which boasts of its large circulation, gathered up all the lying reports about the new movement and put them forth seemingly to show what an unreliable paper the *Sun* was.

When Mr. Owen endeavored to show their errors by quoting from the United States authorities, the editor utterly refused to make things right and kept on with his falsehoods.

Mr. Preston absolutely perverts the whole matter of the Familistere and my account of it. It is a wonderful social development, and he ought to study it and learn something about it. I was showing how *marvelous* it was that nearly 2,000 common laboring people could be enabled, by co-operation, to live in a palace, in their own independent suites of rooms, and have their park, their theatre, their baths, their unequaled schools and nurseries, their library, their festivals, their music, with old age and sickness provided for, and all this without a criminal or pauper in the whole twenty-seven years. Now Mr. Preston comes forward and tries to make out that I considered it marvelous because they got as many as 2,000 persons together! Their aim was not to get large numbers, but simply to supply the iron business with which they were connected. Such successful establishments usually have hundreds, perhaps thousands, applying, more than they can take.

For Mr. Preston's information, I will say that communism and integral co-operation are very different things, as co-operation gives individual liberty and all the advantages of unity of interests besides. The establishments he speaks of were communistic and incorrect in their plan. He thinks "colonization is a splendid scheme," but thinks the race is not ripe for it. For all old fogies who are fast asleep as to what the world is already doing, I must say a few words:

Mr. Herman Schultz established a plan of co-operation, and when he died in 1883, there were 3,500 co-operative societies in Germany, besides thousands more in Austria, Italy, Russia, and Belgium which sprang from his methods. These 3,500 societies did a business of £100,000,000 a year.

The following with reference to English co-operation is condensed from the Westminster *Review:* "There are now 1,350 workingmen's co-operative retail stores, having 920,000 members. * * Last year nearly $15,000,000 were divided, in the shape of profits, among the members. * * The co-operators have a bank of their own, handling more than $80,000,000. There are now 877 retail stores, most of which sent delegates to the recent congress at Dewsbury, by the English Co-operative Wholesale Society, whose sales last year amounted to nearly $30,000,000. The *Wholesale*, as it is called, has a trading capital of about $4,500,000, and among its assets four steamships engaged in carrying goods between England and the Continent."

Thus facts already show overwhelmingly that co-operation is a grand success, and George Jacob Holyoake shows how remarkably it is ennobling the people.

The Nationalistic movement in this country which has already over 200 societies, is co-operative in its nature. They build upon Mr. Bellamy's book, *Looking Backward*, and include some of the finest minds in the United States.

True co-operation makes a brotherhood and sisterhood of all mankind and demands that the strong, the greedy and the cunning shall not trample their fellows under their feet, under this unrighteous system of competition.

---

Since writing the foregoing, Mr. Preston has another article in the JOURNAL. He is still bringing up the old platitudes which signify that people are not good enough to dwell together in co-operative harmony, therefore they should not attempt it, but wait until they are good. In other words, no one must go into the water until he can swim. Stand up for the present competitive system which sets every man against his neighbor, trample the weak under foot and thus allow a very few to get nearly everything in their hands while the many are starving. This is the way to make people good, is it?

One man in San Francisco does not like the Credit Foncier Co. of Sinaloa because he thinks it too much on the plan of a speculation. It *is* in part a speculation, and heaven grant that it may prove a successful one. A grand colony based on a principle of equal wages and rights to women to those which are granted to men, which deals liberally with all its members, caring tenderly for the aged, for the young and for the unfortunate, and dividing the profits of their speculations among all the members, a colony which has to compete with the pauper labor of the old world should have just as many advantages as possible. I glory in the fact that they have such a wonderful bay and harbor right in the best pathway between Asia and our great eastern cities, a fact which must inevitably make them a great commercial port. I have just learned that a syndicate has been formed and capital subscribed to build their railroad immediately after a certain arrangement has been made which fact will no doubt grieve Mr. Preston after his lugubrious talk.

A writer in the *Sun* returned from the colony pretends that the fine accounts of the colony given in their paper, *The Credit Foncier* of Sinaloa, are false. To state that its editor, Mrs. Marie Howland, a lady of national reputation and the highest character would falsify, is too absurd for a moment's notice. I have personally seen so many letters and people from there, that I know these statements are not false.

50 *Union Square, N. Y.*

